# MRI reveals increased superior vena caval blood flow in human fetuses with congenital heart disease, abnormal placental pathology and neonatal brain white matter changes

**DOI:** 10.1186/1532-429X-17-S1-O92

**Published:** 2015-02-03

**Authors:** Sujana Madathil, Liqun Sun, Brahmdeep S Saini, Shi-Joon Yoo, Edgar Jaeggi, Lars Grosse-Wortmann, John Kingdom, Edward J Hickey, Steven Miller, Christopher Macgowan, Mike Seed

**Affiliations:** 1 Paediatric Cardiology, The Hospital for Sick Children, Toronto, ON, Canada; 2 Physiology & Experimental Medicine, The Hospital for Sick Children, Toronto, ON, Canada; 3 Cardiovascular Surgery, The Hospital for Sick Children, Toronto, ON, Canada; 4 Neurology, The Hospital for Sick Children, Toronto, ON, Canada; 5 Obstetrics & Gynecology, Mount Sinai Hospital, Toronto, ON, Canada; 6 Diagnostic Imaging, The Hospital for Sick Children, Toronto, ON, Canada

## Background

Delayed brain development in newborns with congenital heart disease (CHD) results in increased vulnerability to white matter injury before and after cardiac surgery [1]. Doppler showing reductions in pulsatility index (PI) in the middle cerebral arteries (MCA) of fetuses with CHD is interpreted as evidence of "brain sparing physiology". It supports the hypothesis that *in utero* brain dysmaturation in CHD is due to reduced cerebral oxygen delivery [2]. We were interested in the relationship between cerebral blood flow, placental pathology and white matter changes in CHD fetuses using ultrasound and MRI.

## Methods

Late gestation CHD fetuses and normal controls were studied using Doppler and a 1.5T MRI system (Siemens Avanto). We measured the MCA PI with ultrasound, superior vena cava (SVC) flow and fetal brain weight (EBW) with MRI using our previously published technique [3]. Placental histology was studied. Head ultrasound (HUS) performed after delivery was used to classify the newborn brains as: normal, increased white matter echogenicity (WME), or periventricular leukomalacia (PVL). The fetal parameters were compared in babies with and without brain abnormalities using an unpaired Student *t*-test with p<0.05 taken as statistically significant. Odds ratios for abnormal brain imaging, placental abnormalities and SVC flow were calculated.

## Results

**Figure 1:**
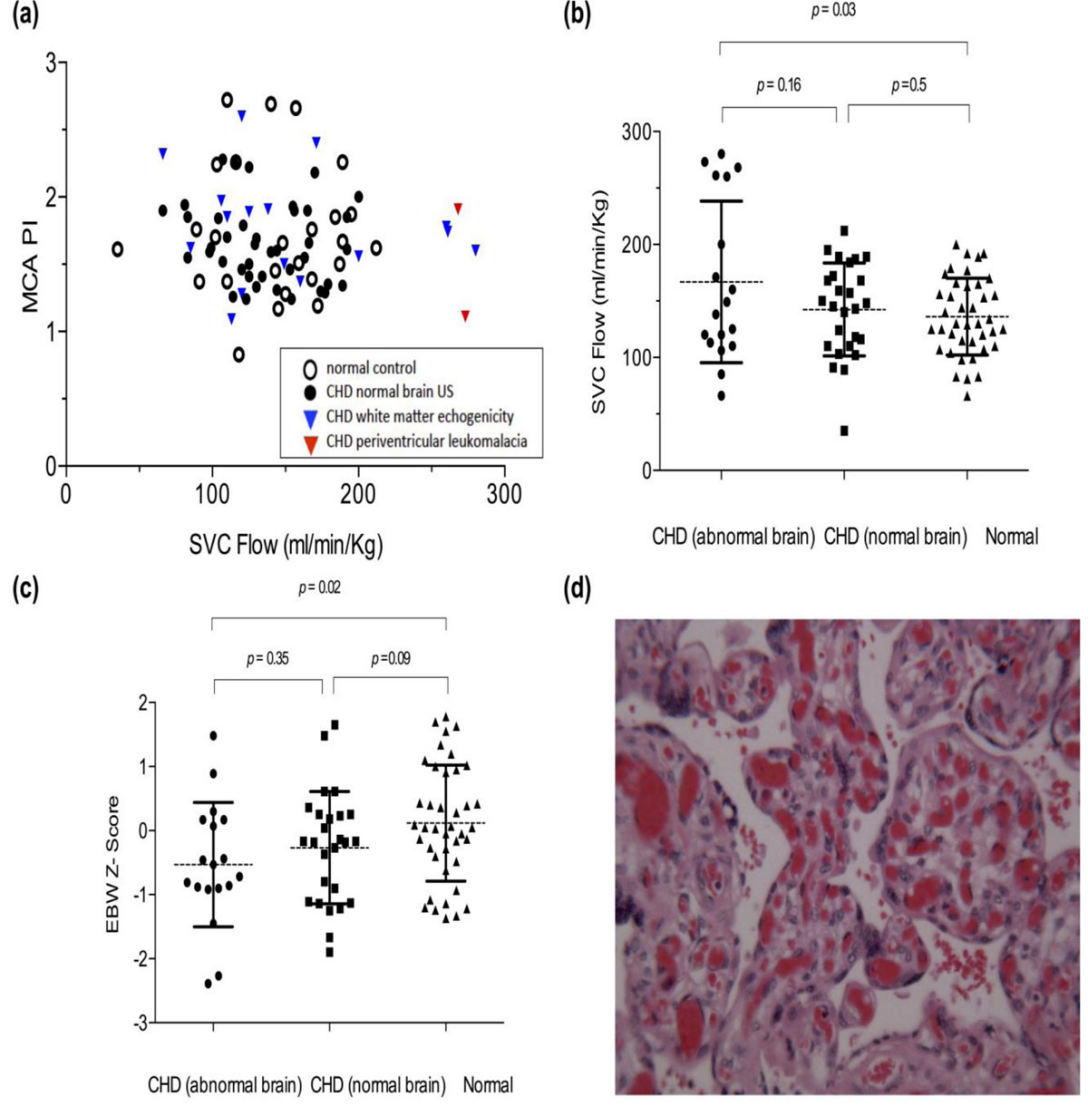
**Comparison of brain ultrasound findings with fetal hemodynamic parameters and placental pathology. (a)** There was no difference in middle cerebral artery pulsatility index (MCA PI) between normal and congenital heart disease (CHD) fetuses with or without white matter changes at birth. **(b)** CHD fetuses with abnormal brains at birth had higher superior vena cava (SVC) flow than normals (p=0.03) and **(c)** smaller brains than those with normal head ultrasound (p = 0.02). **(d)** The histological examination of the placenta of a fetus with aortic coarctation and white matter changes consistent with periventricular leukomalacia showed marked chorangiosis and thrombotic vasculitis.

Forty four fetuses with CHD [mean gestational age (GA): 36weeks, SD 1.4 weeks] and forty normal fetuses (mean GA: 37 weeks, SD 1.3 weeks) were studied. There was no difference in MCA PI between normal and CHD fetuses with or without white matter changes at birth (Fig. 1a, Table 1). 34% of neonates with CHD had increased WME on HUS and 7% had PVL. Fetuses with changes on brain imaging at birth had higher SVC flow than normals (p=0.03) (Fig. 1b) and smaller brains than those with normal HUS (p = 0.05) (Fig. 1c). Elevated SVC flow was associated with a markedly increased risk of PVL (OR: 41, p = 0.005). CHD fetuses with WME had high incidence of histologically abnormal placenta (OR: 6.1, p = 0.04) (Fig. 1d).

**Table 1:** Head ultrasound findings, fetal hemodynamic parameters and placental abnormalities.

	CHD Abnormal Brain	CHD Normal Brain	Normal
**EBW z-score**	-0.53 ± 0.23 (n=18)	-0.27 ± 0.17 (n=26)	0.12 ± 0.14 (n=40)

**SVC Flow (ml/min/kg)**	166.9 ± 16.86 (n=18)	142.5 ± 8.08 (n=26)	136.1 ± 5.38 (n=40)

**MCA PI**	1.75 ± 0.1 (n=18)	1.74 ± 0.1 (n=25)	1.63 ± 0.05 (n=40)

**Placental Abnormalities (%)**	81.3	59.1	N/A

CHD: Congenital heart disease; EBW: Estimated brain weight; SVC: Superior vena cava; MCA PI: Middle cerebral artery pulsatility index

## Conclusions

In keeping with previous studies, we found smaller brains and a high incidence of WME and PVL in newborns with CHD. Abnormally high SVC flow, in keeping with "brain-sparing physiology" appears to be highly associated with PVL, suggesting that the combination of CHD and placental disease may be dangerous for the immature white matter in CHD newborns. Abnormal SVC flow by fetal cardiac MRI may be a more useful indicator of increased risk for white matter injury than MCA PI during late gestation, and could indicate early delivery by Cesarean section.

## Funding

N/A.
